# Distinguishing Organizing Pneumonia from Malignancy During Cancer Surveillance: Predominant Diagnostic Value of Imaging over Systemic Inflammatory Biomarkers

**DOI:** 10.3390/curroncol33080491

**Published:** 2026-08-20

**Authors:** Hacer Boztepe Yesilcay, Asim Armagan Aydin

**Affiliations:** 1 Department of Thoracic Surgery, Antalya Training and Research Hospital, University of Health Sciences, 07100 Antalya, Turkey; 2 Department of Clinical Oncology, Antalya Training and Research Hospital, University of Health Sciences, 07100 Antalya, Turkey

**Keywords:** organizing pneumonia, cancer surveillance, pulmonary recurrence, thoracic oncology, positron emission tomography/computed tomography, inflammatory biomarkers, diagnostic modeling, risk stratification

## Abstract

**Background:** Distinguishing organizing pneumonia from recurrent malignancy in patients undergoing cancer surveillance remains a major clinical challenge. Although inflammatory biomarkers have emerged as potential diagnostic tools, their incremental value beyond imaging-based assessment remains uncertain. **Methods:** In this retrospective single-center study, we evaluated 170 patients with prior malignancy who underwent surgical assessment for suspicious lung-only pulmonary lesions between 2013 and 2023. Histopathology confirmed organizing pneumonia in 61 patients and malignancy in 109 patients. Preoperative clinical, radiologic, Positron emission tomography/computed tomography (PET/CT), and inflammatory biomarker variables were incorporated into predefined clinical, radiology-PET/CT, biomarker, and integrated models using Least absolute shrinkage and selection operator (LASSO)-penalized logistic regression with internal cross-validation. **Results:** The radiology/PET model demonstrated excellent discrimination (AUC 0.927), closely approximating the performance of the integrated model (AUC 0.935). The clinical and biomarker-only models showed moderate discrimination (AUC 0.764 and 0.778, respectively). Adding inflammatory biomarkers to imaging-derived features resulted in only a minimal improvement in diagnostic performance (ΔAUC = 0.009, 95% CI −0.019 to 0.037), indicating no statistically significant incremental benefit. Calibration, decision curve, and predictor stability analyses consistently identified radiologic and PET/CT-derived variables as the principal sources of predictive information. **Conclusions:** In patients with suspicious pulmonary lesions during cancer surveillance, diagnostic discrimination was driven predominantly by imaging-derived features, whereas conventional inflammatory biomarkers provided limited incremental value. These findings support an imaging-centered approach to preoperative risk stratification and multidisciplinary decision-making.

## Simple Summary

Patients with a history of cancer often develop new pulmonary lesions during follow-up, making it difficult to distinguish recurrent malignancy from benign inflammatory conditions such as organizing pneumonia. In this study, we compared the diagnostic contributions of clinical characteristics, imaging findings, PET/CT features, and inflammatory biomarkers to preoperative lesion assessment. Radiologic and PET/CT variables provided most of the discriminative information, whereas inflammatory biomarkers added only limited incremental value. Overall, our findings emphasize the central role of imaging-derived features in this diagnostic setting and offer a foundation for future studies aimed at developing and validating clinically applicable prediction models.

## 1. Introduction

Distinguishing recurrent malignancy from benign inflammatory pulmonary lesions remains a major diagnostic challenge in thoracic oncology [1,2]. In patients with a previous history of cancer, newly detected pulmonary lesions are often considered suspicious for recurrence, particularly when they exhibit concerning morphologic or metabolic imaging features [3,4]. As a result, many patients undergo invasive biopsy or surgical resection to establish a definitive histopathological diagnosis [5,6]. However, a substantial proportion of these lesions are ultimately found to represent benign inflammatory conditions, most commonly organizing pneumonia, leading to unnecessary invasive procedures, procedure-related morbidity, and delays in subsequent clinical management [7,8,9].

Organizing pneumonia is characterized by intra-alveolar fibroblastic plugs accompanied by inflammatory cell infiltration and abnormal tissue repair [10]. Because its radiologic appearance frequently overlaps with that of recurrent malignancy, differentiation between the two entities can be difficult [11]. On computed tomography (CT), organizing pneumonia may present with solid nodules or masses, spiculation, cavitation, multifocal lesions, or irregular margins, all of which can mimic malignant disease [12]. Likewise, inflammatory activity may result in increased ^18F-FDG uptake on positron emission tomography/computed tomography (PET/CT), further reducing diagnostic specificity in patients with a high pretest probability of recurrence [8,13,14].

Blood-based inflammatory biomarkers have attracted increasing interest as readily available indicators of systemic inflammation and the host response to cancer [15]. Composite indices such as the systemic immune-inflammation index (SII) [16], monocyte-to-lymphocyte ratio (MLR) [17], C-reactive protein-to-albumin ratio (CAR) [18], lactate dehydrogenase-to-albumin ratio (LAR) [19], and global immune-nutrition-inflammation index (GINI) [20] have been associated with prognosis and treatment outcomes in several malignancies. These biomarkers are thought to reflect cancer-related inflammatory activation, metabolic alterations, immune imbalance, and overall physiological status [21]. However, benign inflammatory pulmonary diseases can trigger many of the same systemic responses, making it unclear whether these biomarkers provide additional diagnostic information beyond conventional imaging [22].

Most previous studies addressing this diagnostic problem have evaluated imaging findings or inflammatory biomarkers in isolation, often using univariable approaches or small retrospective cohorts [23]. Consequently, the relative contributions of clinical variables, radiologic and PET/CT-derived features, and systemic inflammatory biomarkers within integrated preoperative prediction frameworks remain poorly defined [24]. Moreover, whether inflammatory biomarkers provide meaningful incremental diagnostic value beyond imaging-centered assessment has not been systematically evaluated using robust internal validation and multidimensional model performance analysis [25,26].

Accordingly, we conducted a retrospective diagnostic modeling study in patients with prior malignancy who underwent surgical evaluation for suspicious pulmonary lesions during oncologic follow-up. Using exclusively preoperative clinical, radiologic, PET/CT, and blood-based inflammatory variables, we developed and internally validated multidomain prediction models to distinguish organizing pneumonia from malignancy. We further assessed discrimination, calibration, clinical utility, and predictor stability to define the relative diagnostic contribution of imaging-derived and inflammatory features within a clinically applicable preoperative framework.

## 2. Materials and Methods

### 2.1. Study Design and Patient Selection

This retrospective diagnostic modeling study included patients with a prior history of localized or locally advanced malignancy who developed pulmonary lesions suspicious for recurrence during oncologic follow-up between May 2013 and September 2023. All patients had previously undergone curative-intent treatment, including surgical resection with or without indicated adjuvant therapy, and had achieved at least 2 years of recurrence-free follow-up before detection of the pulmonary lesion.

Patients underwent surgical resection for radiologically suspected lung-only recurrence at the Thoracic Surgery Department of a tertiary referral center. Only cases with histopathologically confirmed organizing pneumonia or malignancy were included in the final analytic cohort, with organizing pneumonia constituting the non-malignant group and malignant lesions serving as the comparator group. All malignant lesions were confirmed as pulmonary recurrence or metastatic involvement of previously diagnosed extrathoracic malignancies based on histopathological evaluation. No primary lung cancer cases were identified in the malignant cohort.

To establish a clinically homogeneous diagnostic setting, patients with concurrent extrapulmonary metastatic disease were excluded. Additional exclusions included absence of definitive pathological diagnosis, incomplete clinical, imaging, or laboratory data, loss to follow-up during diagnostic evaluation, absence of preoperative PET/CT imaging, administration of systemic antibiotics or corticosteroids within 4 weeks before laboratory assessment, blood transfusion before surgery, and conditions potentially influencing inflammatory biomarker interpretation.

All patients underwent contrast-enhanced thoracic computed tomography (CT) with 2 mm slice thickness and fluorodeoxyglucose positron emission tomography/computed tomography (PET/CT) before surgical decision-making. PET/CT examinations were performed using a Siemens PET/CT system according to the institutional standard oncologic imaging protocol. Patients fasted for at least 6 h before FDG administration, and PET/CT imaging was performed approximately 60 min after intravenous FDG injection. Thoracic CT, PET/CT, and peripheral blood laboratory tests were obtained as part of the same routine preoperative diagnostic work-up before surgical decision-making. Peripheral blood samples for inflammatory biomarker analysis were collected within 7 days before surgery.

Only variables available before pathological diagnosis and surgical intervention were incorporated into model development to minimize information leakage. Postoperative pathological staging and surgery-related variables were excluded from all analyses.

**Figure 1:**
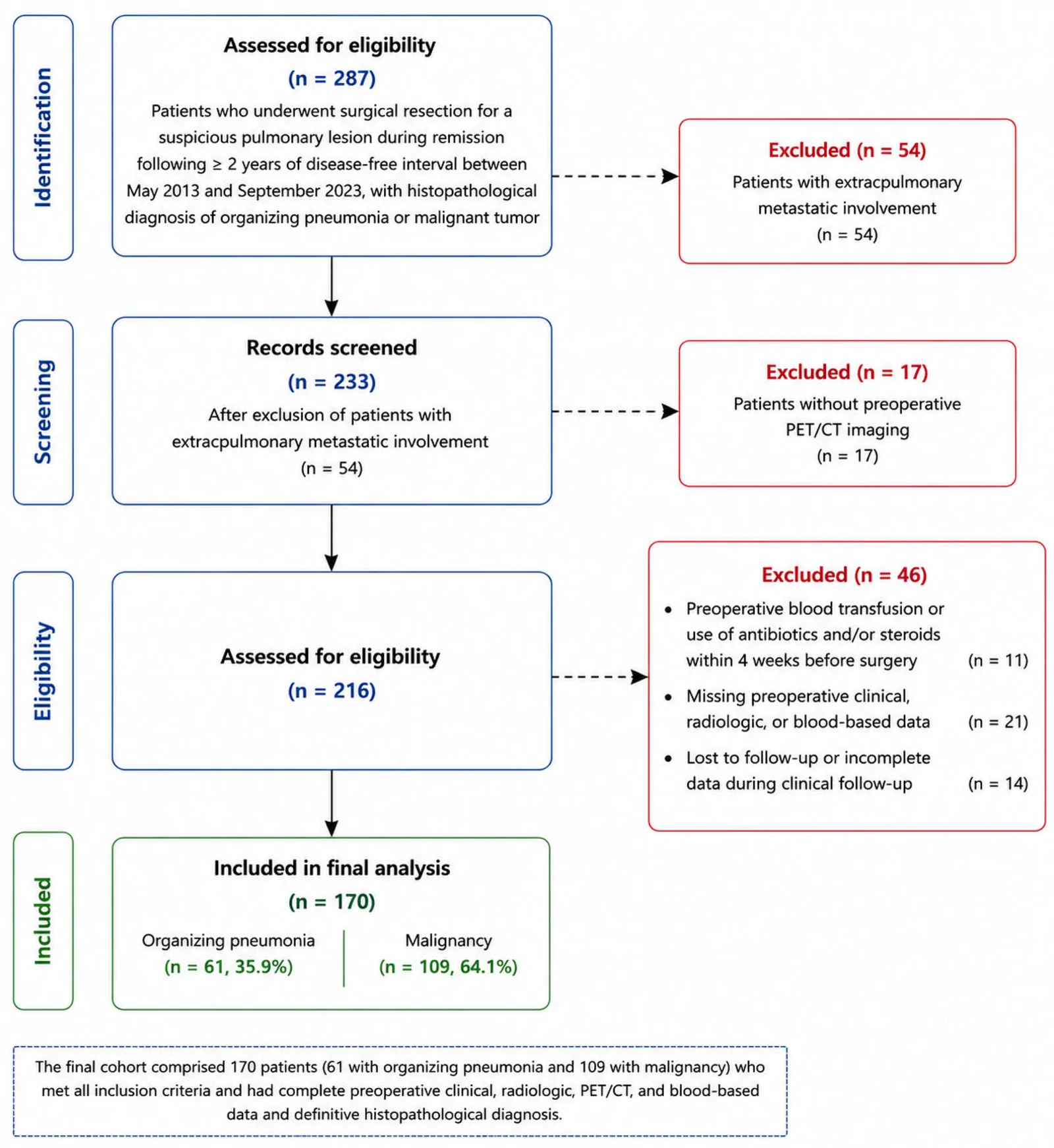
Study Flow Diagram and Patient Selection. **Abbreviations:** PET/CT, positron emission tomography/computed tomography.

The final analytic cohort consisted of 170 patients, including 61 with organizing pneumonia and 109 with malignancy. Patient selection and cohort allocation are summarized in Figure 1.

### 2.2. Data Collection and Candidate Predictors, and Outcome Definition

Clinical, radiological, PET/CT, and laboratory variables were retrospectively extracted from institutional electronic medical records and imaging archives using a predefined analytical framework. Chest CT and PET/CT images were retrospectively reviewed by two thoracic radiologists with experience in thoracic imaging. Radiologists were blinded to the final histopathological diagnosis and laboratory data but were aware of the clinical indication of oncologic surveillance. Discrepancies between readers were resolved by consensus. Because this was a retrospective analysis of routinely acquired clinical imaging data, formal interobserver agreement analysis was not available. Imaging findings were classified according to predefined study criteria for subsequent analysis.

Clinical data included age, sex, Eastern Cooperative Oncology Group performance status (ECOG PS), smoking history, comorbidity status, and the presence of symptoms at presentation. Radiologic variables comprised lesion diameter, lesion location, multifocality or satellite lesions, CT morphology, cavitation, spiculation, air bronchogram, and the presence of a halo or reverse halo sign. Lesion diameter and PET/CT-derived SUVmax values were analyzed as continuous variables. CT morphology was classified into four categories (solid, consolidation, ground-glass, and mixed). Cavitation, spiculation, air bronchogram, mediastinal lymph node involvement, halo/reverse halo sign, and multifocality/satellite lesions were recorded as binary variables (present/absent). Mediastinal lymph node involvement was defined as increased FDG uptake relative to background mediastinal activity and/or suspicious morphologic enlargement on CT. Multifocality was defined as the presence of multiple pulmonary lesions. Cavitation was defined as an air-containing lucent area within the pulmonary lesion. Spiculation was defined as linear strands extending from the lesion margin into the surrounding lung parenchyma. Halo and reverse-halo signs were assessed according to established radiological definitions.

All patients underwent preoperative fluorodeoxyglucose positron emission tomography/computed tomography (PET/CT) scans. The PET/CT-derived variables included the maximum standardized uptake value (SUVmax) of the primary pulmonary lesion, mediastinal lymph node involvement, and SUVmax of the largest mediastinal lymph node. The unit of analysis was the patient. In patients with multiple pulmonary lesions, imaging characteristics, including lesion diameter, CT morphology, cavitation, spiculation, and PET/CT parameters, were recorded from the surgically resected index lesion, which served as the reference lesion for the final histopathological diagnosis.

Peripheral blood-based inflammatory biomarkers obtained within 7 days before surgery included hemoglobin, neutrophil-to-lymphocyte ratio (NLR) [17], monocyte-to-lymphocyte ratio (MLR) [17], platelet-to-lymphocyte ratio (PLR) [17], pan-immune-inflammation value (PIV) [27], systemic immune-inflammation index (SII) [16], systemic inflammation response index (SIRI) [28], C-reactive protein-to-albumin ratio (CAR) [18], lactate dehydrogenase-to-albumin ratio (LAR) [19], and global immune-nutrition-inflammation index (GINI) [20].

The primary objective of this study was to develop preoperative predictive models capable of distinguishing organizing pneumonia from malignancy in patients with radiologically suspicious pulmonary lesions during oncologic follow-up. The binary study outcome was defined according to the final histopathological diagnosis obtained after surgical resection.

### 2.3. Statistical Analysis

Continuous variables are summarized as medians and interquartile ranges (IQR), and categorical variables as frequencies and percentages. Between-group comparisons were performed using the Mann–Whitney U, χ^2^, or Fisher’s exact tests, as appropriate. Missing data were addressed using multiple imputation by chained equations under the missing-at-random assumption. Ten imputed datasets were created, and predictor stability was evaluated across these datasets. To avoid information leakage, imputation was carried out separately within each training fold during repeated 5-fold cross-validation.

Before model development, the analytical framework was prespecified to compare the diagnostic contributions of four predictor domains: clinical variables, radiology/PET variables, inflammatory biomarkers, and their combination. Although no prospective preregistered statistical analysis plan was available because of the retrospective study design, the multidomain modeling framework was defined before model fitting on the basis of the study objectives, clinical rationale, and previous literature, and was not modified according to the observed model performance.

To compare the diagnostic value of different data domains, four predictive models were developed based on clinical, radiologic/PET, inflammatory biomarker, and integrated multidomain variables. Candidate predictors included 5 clinical, 8 radiologic, 3 PET/CT, and 10 inflammatory biomarker variables, selected a priori according to clinical relevance and previous literature. Model development was performed using LASSO-penalized logistic regression, with the optimal penalty parameter (λ) determined by cross-validation. Only predictors with non-zero coefficients were retained in the final models.

Internal validation was conducted using repeated 5-fold cross-validation with out-of-fold predictions. Missing data were imputed separately within each training fold to avoid information leakage.

Model performance was evaluated using the area under the receiver operating characteristic curve (AUC). Sensitivity, specificity, ΔAUC with 95% confidence intervals, calibration (calibration plots, calibration slope, and Brier score), decision curve analysis, and predictor selection frequency across imputed datasets were also assessed.

All statistical analyses were performed using R software (version 4.4.1; R Foundation for Statistical Computing, Vienna, Austria) and Python (version 3.11; Python Software Foundation, Wilmington, DE, USA). Statistical significance was set at *p* < 0.05.

### 2.4. Ethical Approval

The study was conducted in accordance with the ethical principles of the Declaration of Helsinki and approved by the Ethics Committee of the University of Health Sciences Antalya Training and Research Hospital (approval number: 4/16; 19 February 2026). Owing to the retrospective design and use of anonymized clinical data, the requirement for informed consent was waived by the institutional review board.

## 3. Results

### 3.1. Study Population and Baseline Characteristics

**Figure 2:**
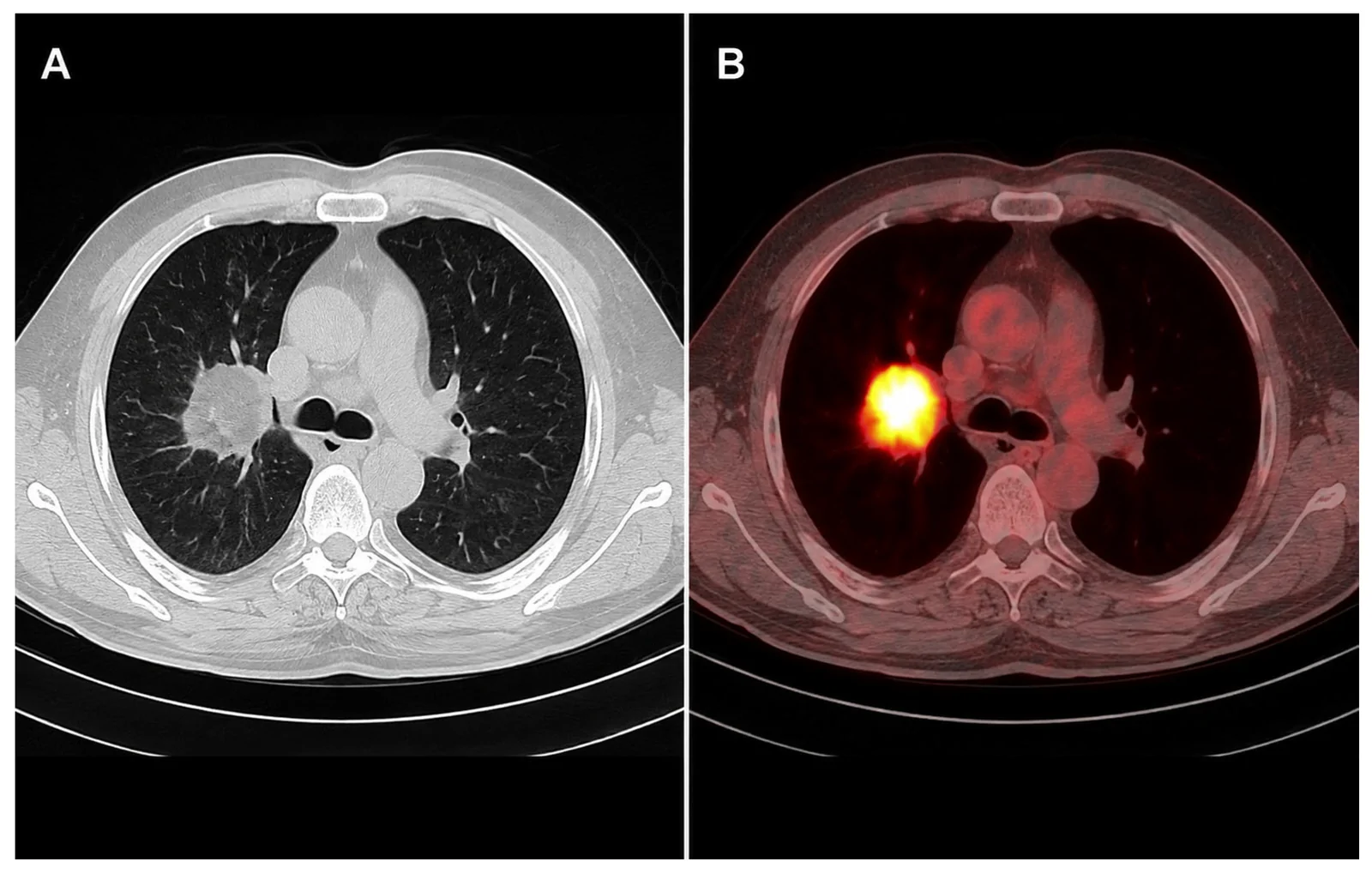
Representative CT and PET/CT findings. (**A**) Contrast-enhanced thoracic CT demonstrating a solitary pulmonary lesion suspicious for recurrent malignancy during oncologic follow-up. (**B**) Corresponding fused ^18F-FDG PET/CT image showing intense FDG uptake within the lesion, illustrating the imaging characteristics evaluated in the radiology/PET prediction model. **Abbreviations:** PET/CT, Positron emission tomography/computed tomography; FDG, Fluorodeoxyglucose.

A total of 170 patients were included in the analytic cohort, comprising 61 patients with organizing pneumonia and 109 with malignancy. All patients had a history of prior malignancy in remission and underwent surgical resection for radiologically suspected recurrent pulmonary lesions. Only preoperative variables available before surgical decision-making were included in the analyses. Postoperative and surgery-related variables were excluded a priori to prevent data leakage and ensure the development of a clinically applicable diagnostic model. Representative preoperative CT and corresponding ^18F-FDG PET/CT images of a pulmonary lesion evaluated in the study cohort are shown in Figure 2.

Baseline demographic, clinical, oncologic, radiologic, PET/CT, and laboratory characteristics are summarized in Table 1. The distribution of antecedent primary malignancies did not differ significantly between patients with organizing pneumonia and those with histopathologically confirmed malignant pulmonary lesions (*p* = 0.143). Colorectal cancer was the most common primary tumor in both groups (26/61 [42.6%] vs. 48/109 [44.0%]), followed by breast (9/61 [14.8%] vs. 20/109 [18.3%]), genitourinary (11/61 [18.0%] vs. 16/109 [14.7%]), gynecologic (6/61 [9.8%] vs. 10/109 [9.2%]), and other malignancies (9/61 [14.8%] vs. 15/109 [13.8%]), respectively. Among patients with malignant pulmonary lesions, 86/109 (78.9%) had previously received chemotherapy and 40/109 (36.7%) had received radiotherapy. Radiologic characteristics differed between the diagnostic groups, including lesion morphology, multifocality or satellite lesions, cavitation, spiculation, and halo or reverse-halo signs. Blood-based inflammatory indices also differed between groups, although their distributions showed substantial overlap.

### 3.2. Differences in Preoperative Clinical, Radiologic, and Biomarker Features

**Figure 3:**
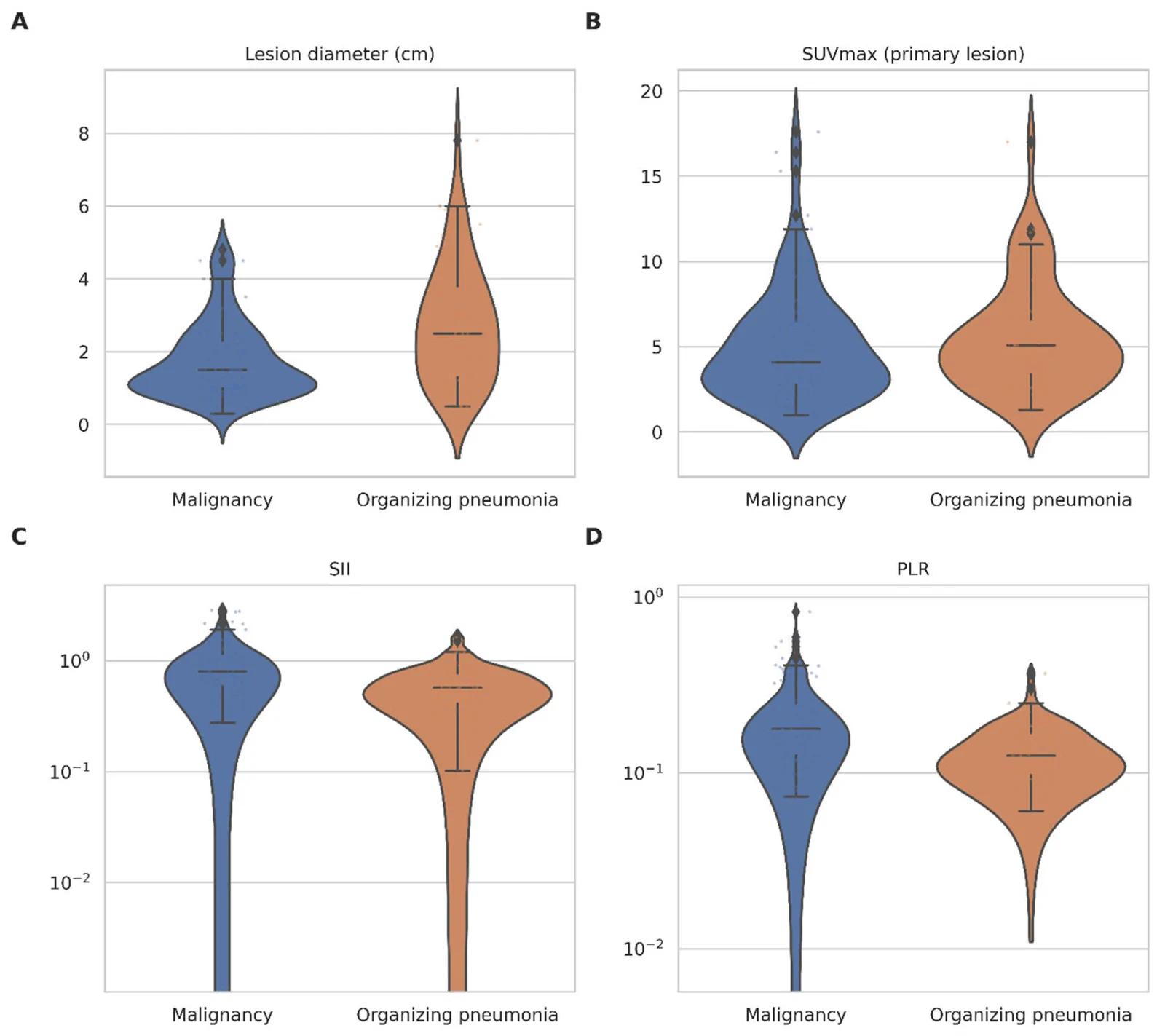
Distribution of preoperative radiologic and inflammatory biomarkers according to final diagnosis. Violin plots with overlaid boxplots and individual data points illustrate the distribution of key preoperative variables stratified by organizing pneumonia and malignancy. Radiologic features, including lesion diameter (**A**) and PET/CT-derived SUVmax (**B**), demonstrated clear separation between groups. In contrast, blood-based inflammatory indices, such as SII (**C**) and PLR (**D**), showed overlapping distributions despite moderate differences in central tendency. Logarithmic scaling was applied to biomarker variables to account for skewed distributions. These findings support the dominant role of radiologic and metabolic features in discriminating malignancy from organizing pneumonia. **Abbreviations:** PET/CT, positron emission tomography/computed tomography; SUVmax, maximum standardized uptake value.

Significant differences between organizing pneumonia and malignancy were primarily observed in the radiologic and PET/CT-derived variables. Lesion morphology, including CT pattern, cavitation, multifocality, and halo or reverse halo signs, differed substantially between the groups. In addition, lesion diameter and PET/CT metabolic activity demonstrated a strong discriminatory potential. In contrast, blood-based inflammatory indices, including the SII, PLR, CAR, and LAR, showed only moderate between-group differences with considerable overlap in distribution. These findings suggest that radiologic and metabolic imaging features are the principal sources of diagnostic information in this clinical setting (Figure 3).

### 3.3. Model Development and İnternal Validation

Four diagnostic models were predefined to distinguish malignancy from organizing pneumonia: a clinical model, a radiology/PET model, a biomarker model, and an integrated model incorporating all preoperative predictors. Model development was based on LASSO-penalized logistic regression. Internal validation was performed using cross-validated out-of-fold predictions and repeated 5-fold cross-validation to assess model stability and reduce the risk of overfitting.

### 3.4. Model Discrimination Performance

**Figure 4:**
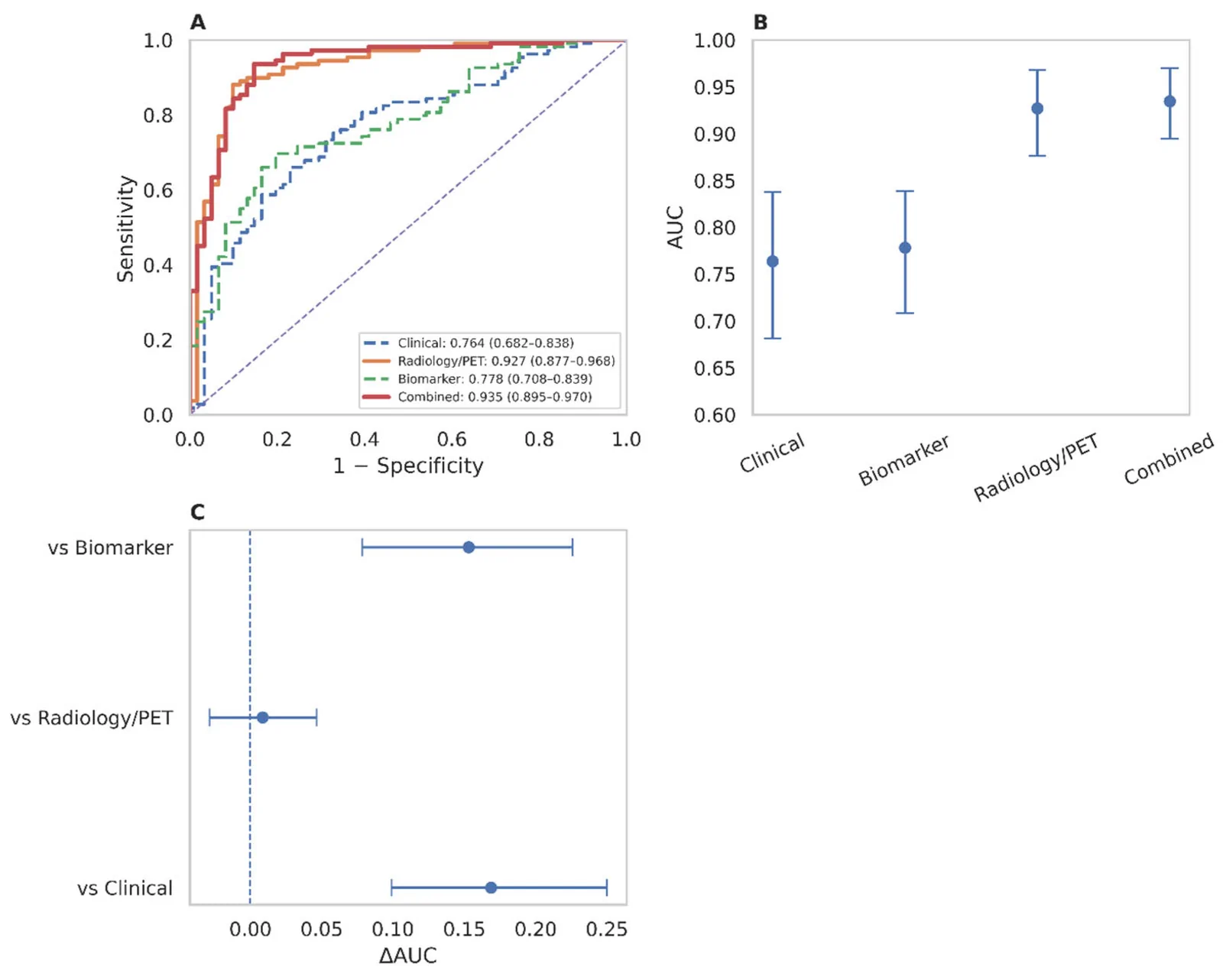
Discriminative performance and incremental value of preoperative prediction models. (**A**) Cross-validated receiver operating characteristic (ROC) curves for the clinical, radiology/PET, biomarker, and combined models. (**B**) Out-of-fold area under the curve (AUC) estimates with 95% confidence intervals. (**C**) Differences in AUC (ΔAUC) comparing the combined model with individual component models. The radiology/PET model demonstrated strong discrimination, while the combined model achieved the highest overall performance. However, the incremental gain from adding blood-based biomarkers to imaging features was limited. ROC curves and performance metrics were derived from cross-validated out-of-fold predictions. AUC confidence intervals were estimated using bootstrap resampling. ΔAUC values represent differences in discrimination between models and are presented with 95% confidence intervals. Footnote: In panel (**A**), the diagonal dashed line represents the no-discrimination reference (AUC = 0.50); in panel (**C**), the vertical dashed line denotes the null reference (ΔAUC = 0). PET, Positron emission tomography.

The discriminative performances of the four predefined models are summarized in Table 2 and Figure 4.

The clinical and biomarker-only models demonstrated moderate discrimination, with AUCs of 0.764 and 0.778, respectively. In contrast, the radiology/PET model achieved excellent discrimination (AUC = 0.927), with balanced sensitivity (0.881) and specificity (0.902). The integrated model showed the highest discriminative performance (AUC = 0.935), with improved sensitivity (0.936) and a modest reduction in specificity (0.852) (Table 2).

The receiver operating characteristic (ROC) curves showed substantial overlap between the integrated and radiology/PET models, indicating similar discriminative performance (Figure 4A). This was further supported by the AUC estimates and their 95% confidence intervals shown in Figure 4B.

### 3.5. Incremental Value of Biomarker İntegration

The addition of radiologic and PET/CT features to the clinical model substantially improved discrimination (ΔAUC = 0.169, 95% CI 0.093–0.245). Likewise, the combined model showed better discrimination than the biomarker-only model (ΔAUC = 0.153, 95% CI 0.073–0.223). In contrast, adding inflammatory biomarkers to the radiology/PET model resulted in only a marginal increase in discrimination (ΔAUC = 0.009, 95% CI −0.019 to 0.037), indicating no statistically significant improvement because the confidence interval included zero (Figure 4C). Overall, these findings suggest that most of the predictive information was provided by radiologic and PET/CT variables, whereas inflammatory biomarkers offered limited incremental diagnostic value.

### 3.6. Model Calibration and Overall Accuracy

**Figure 5:**
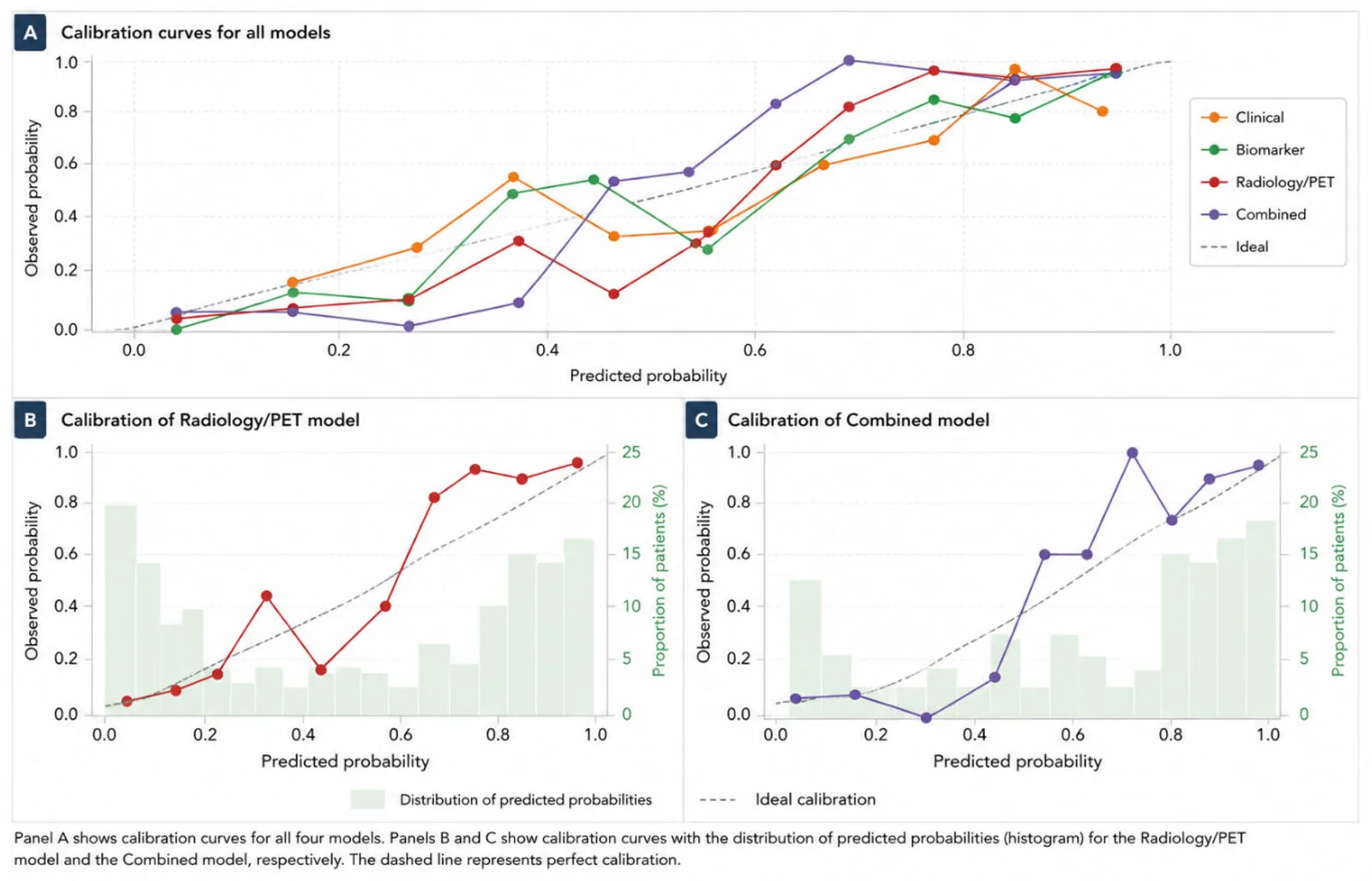
Calibration performance of the diagnostic prediction models. (**A**) Calibration curves for the clinical, biomarker, radiology/PET, and combined models, illustrating agreement between predicted and observed probabilities of malignancy, (**B**) Detailed calibration plot for the radiology/PET model, (**C**) Detailed calibration plot for the combined model. Histograms in Panels (**B**,**C**) represent the distribution of predicted probabilities across the study cohort. Calibration curves were derived from cross-validated out-of-fold predictions. The dashed diagonal line represents perfect calibration, and plotted points correspond to observed event rates within quantiles of predicted risk. PET, Positron emission tomography.

Calibration analysis demonstrated good agreement between the predicted and observed probabilities for the radiology/PET and combined models (Figure 5). The combined model showed a calibration slope close to unity and a low Brier score. Clinical and biomarker-only models demonstrated comparatively poorer calibration and higher prediction errors.

### 3.7. Clinical Utility and Decision Curve Analysis

**Figure 6:**
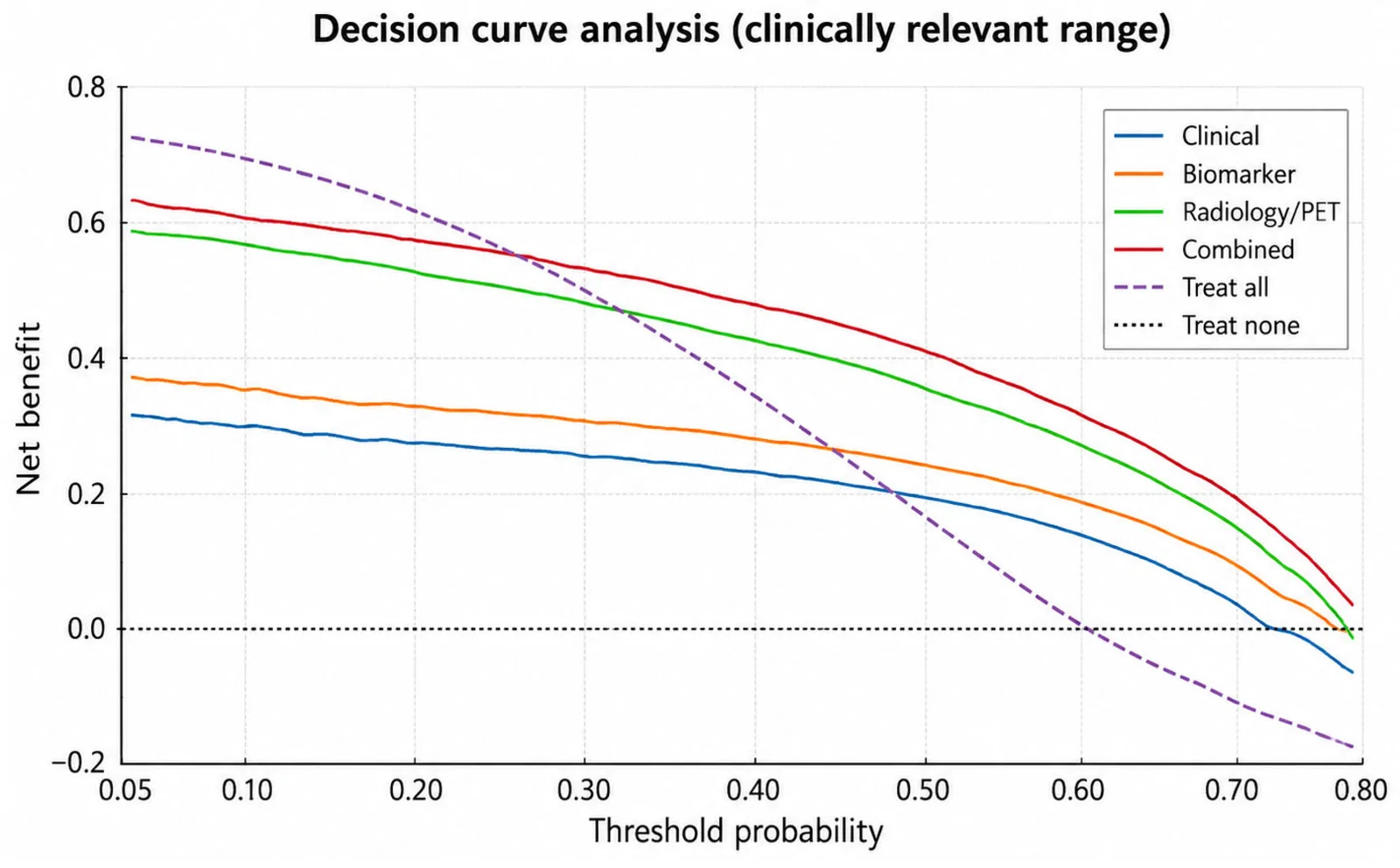
Clinical utility of preoperative prediction models assessed by decision curve analysis. Decision curve analysis demonstrated the net clinical benefit of the clinical, biomarker, radiology/PET, and combined models across clinically relevant threshold probabilities for predicting malignancy. The radiology/PET and combined models consistently provided a greater net benefit than the clinical and biomarker-only models across a broad range of decision thresholds. The reference strategies of “treat all” and “treat none” are shown for comparison. The net benefit estimates were derived from cross-validated out-of-fold predictions. Decision curves were generated across threshold probabilities, reflecting varying clinical risk tolerances for surgical intervention. PET, Positron emission tomography.

Decision curve analysis showed that all prediction models provided a positive net benefit across clinically relevant threshold probabilities (Figure 6). The radiology/PET and integrated models demonstrated nearly overlapping decision curves throughout this range, indicating comparable clinical utility. In contrast, the clinical and biomarker-only models consistently yielded lower net benefit. Because the treat-all strategy showed a marked decline in net benefit at very high threshold probabilities, the clinically relevant threshold range was used for interpretation.

The integrated and radiology/PET models showed similar net benefit across clinically relevant threshold probabilities, whereas the clinical and biomarker-only models performed less favorably. These findings reinforce the predominant contribution of imaging-derived variables to preoperative diagnostic discrimination.

### 3.8. Key Predictors İdentified by LASSO

**Figure 7:**
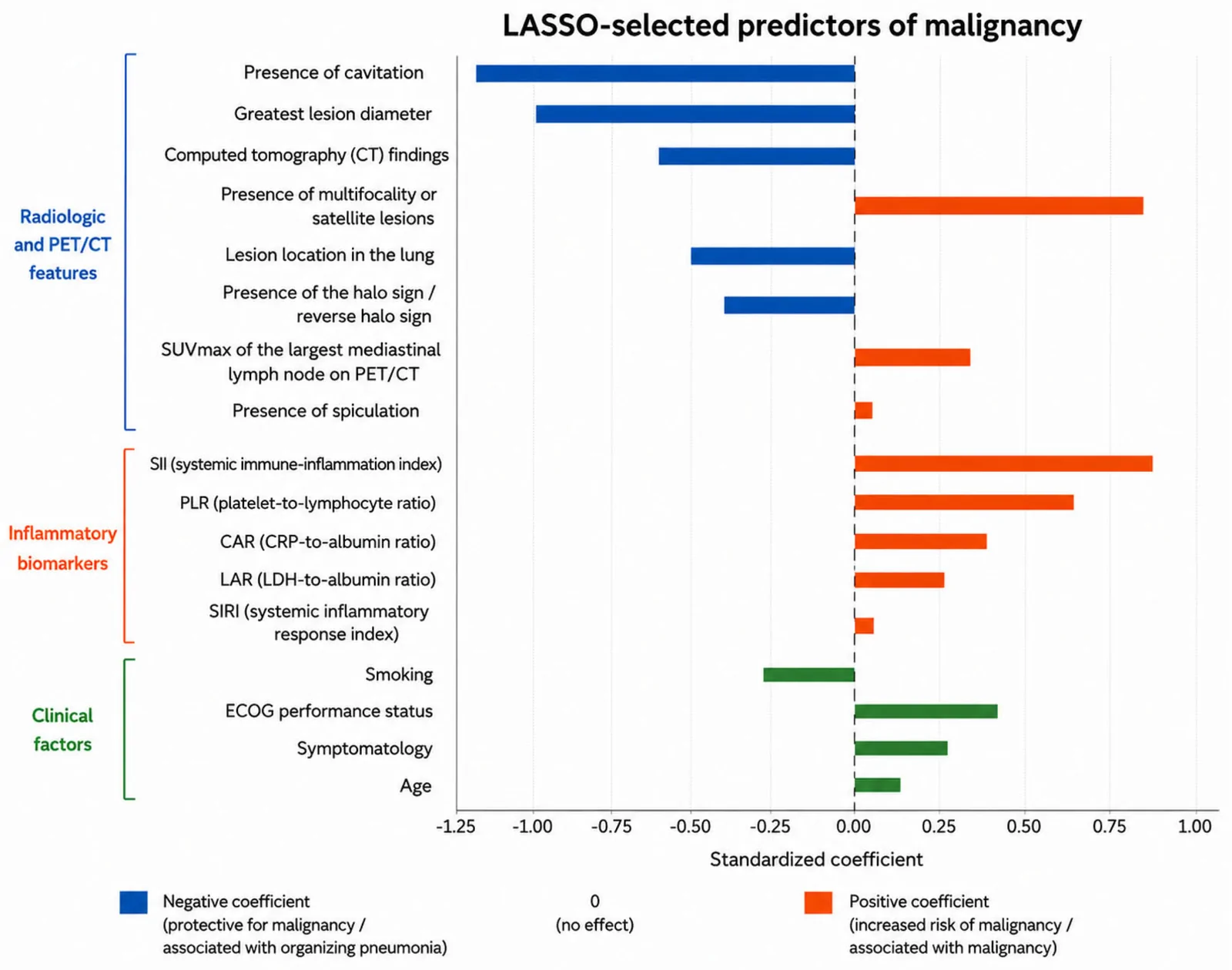
Key predictors retained in the penalized multidomain diagnostic model. Standardized coefficients from the LASSO-penalized logistic regression model identifying the principal predictors of malignancy. Radiologic and PET/CT-derived variables demonstrated the strongest contribution to model discrimination, whereas inflammatory biomarkers and clinical variables showed comparatively smaller effect sizes. The coefficients were estimated using penalized logistic regression with least absolute shrinkage and selection operator (LASSO) regularization. Only variables retained in the final integrated model are shown. Positive coefficients indicate an increased probability of malignancy, whereas negative coefficients indicate a greater association with organizing pneumonia. Blue, orange, and green bars denote radiologic/PET-CT features, inflammatory biomarkers, and clinical factors, respectively; the direction of the bars relative to zero indicates the sign of the standardized coefficient. **Abbreviations:** ECOG, Eastern Cooperative Oncology Group; PET/CT, positron emission tomography/computed tomography; SUVmax, maximum standardized uptake value; CRP, C-reactive protein; LDH, lactate dehydrogenase.

In the penalized regression model, the strongest predictors of malignancy were radiologic variables, including cavitation, lesion diameter, CT pattern, multifocality, and halo or reverse-halo sign (Figure 7).

Blood-based biomarkers, including SII, PLR, CAR, and LAR, were retained with smaller effect sizes, whereas clinical variables contributed modestly to the overall model performance.

## 4. Discussion

In this retrospective diagnostic modeling study, we developed and internally validated an integrated preoperative prediction framework to distinguish organizing pneumonia from malignancy in patients with a prior history of cancer presenting with suspicious pulmonary lesions. Although the combined model incorporating clinical, radiologic, PET/CT, and inflammatory biomarkers demonstrated excellent discriminative performance, the principal and clinically most relevant finding of this study is that the dominant source of predictive information originated from radiologic and PET/CT-derived features, whereas blood-based inflammatory biomarkers provided only limited incremental value beyond imaging.

Distinguishing inflammatory pulmonary lesions from recurrent malignancies remains one of the most challenging scenarios in thoracic oncology [24,29]. Patients with a history of malignancy are frequently evaluated with a high pretest probability of recurrence, often resulting in an aggressive diagnostic and therapeutic approach. However, inflammatory conditions such as organizing pneumonia may closely mimic malignant diseases both radiologically and metabolically, leading to substantial diagnostic uncertainty [12,13]. In clinical practice, this uncertainty frequently culminates in surgical intervention for definitive diagnosis, despite a proportion of lesions ultimately proving to be benign [30]. Consequently, the development of accurate preoperative risk stratification strategies represents an important unmet clinical need in this field.

Although organizing pneumonia represented a relatively frequent benign diagnosis in our cohort, this finding reflects the selected clinical context rather than the prevalence of organizing pneumonia among all patients undergoing oncologic surveillance. Our study population consisted of highly selected patients with previous malignancy who developed pulmonary lesions considered sufficiently suspicious to require invasive diagnostic evaluation. In this setting, organizing pneumonia represents an important benign mimic of recurrent malignancy because of overlapping radiological and metabolic characteristics, including increased FDG uptake on PET/CT. Therefore, our comparison was designed to address a clinically relevant diagnostic dilemma in thoracic oncology: distinguishing benign inflammatory lesions from malignant recurrence before definitive pathological confirmation.

Previous studies have demonstrated that certain CT characteristics, including lesion morphology, spiculation, cavitation, multifocality, and distribution patterns, may assist in differentiating inflammatory lesions from malignancies [1,5,9]. Similarly, PET/CT-derived metabolic parameters, particularly SUVmax, are widely incorporated into clinical decision-making algorithms because of their ability to reflect tumor metabolic activity in patients with cancer [13]. However, inflammatory lesions may also exhibit increased fluorodeoxyglucose uptake, limiting the specificity of PET/CT in isolation [31,32]. In parallel, increasing attention has been directed toward blood-based inflammatory biomarkers as accessible and low-cost indicators of tumor-host interactions, systemic inflammation, and metabolic stress [15,22]. However, most prior investigations have evaluated imaging findings or inflammatory biomarkers separately, often using univariate or limited multivariate approaches. In contrast, the present study systematically integrated multidomain preoperative variables within a rigorously validated predictive modeling framework.

Our findings show that radiologic and PET/CT features provide excellent discrimination, with the radiology/PET model performing similarly to the integrated model. Adding inflammatory biomarkers, including SII, PLR, CAR, and LAR, resulted in only a small improvement in model performance. This pattern was consistent across discrimination, calibration, decision curve, and predictor stability analyses. Although these biomarkers may reflect biologically relevant systemic responses, they contributed little additional diagnostic information beyond imaging in distinguishing malignancy from organizing pneumonia.

From a translational perspective, these findings are biologically plausible. Organizing pneumonia is characterized by intense inflammatory activation, intra-alveolar fibroblastic proliferation, cytokine signaling, and immune cell infiltration, all of which may substantially influence systemic inflammatory responses [33]. Consequently, biomarkers such as SII, PLR, CAR, and LAR may be elevated in both malignant and inflammatory conditions, reducing their specificity for malignancy [25]. In contrast, radiologic and metabolic imaging features more directly reflect lesion-specific biological behaviors, including tumor architecture, spatial growth patterns, tissue invasion, necrosis, and metabolic reprogramming [23]. Features such as spiculation, lesion morphology, cavitation, and increased PET/CT uptake may therefore provide a more direct representation of tumor-intrinsic characteristics than systemic inflammatory signatures [29,31].

Our findings also illustrate the difference between statistical significance and predictive value. Although several inflammatory biomarkers differed significantly between organizing pneumonia and malignancy at the group level, their distributions overlapped substantially, limiting their ability to discriminate between individual patients. This finding highlights that statistically significant associations do not necessarily translate into clinically useful predictive models. It also emphasizes the importance of evaluating candidate biomarkers within multivariable models rather than relying on individual associations alone.

Beyond statistical discrimination, the clinical value of a prediction model ultimately depends on its ability to improve decision making [34]. Decision curve analysis demonstrated that both the radiology/PET and combined models provided a meaningful net clinical benefit across a broad range of threshold probabilities. These findings suggest that imaging-driven predictive models may support multidisciplinary evaluations and improve preoperative risk stratification in patients with suspected recurrent pulmonary lesions. Clinically, such approaches may contribute to reducing unnecessary surgical resections, procedure-related morbidity, and diagnostic delays in patients with benign inflammatory lesions while preserving timely interventions for patients with true malignant recurrence.

The primary intended clinical role of the proposed models is to support preoperative multidisciplinary decision-making in patients with suspicious pulmonary lesions during oncologic surveillance after previous malignancy. The models are not intended to replace histopathological confirmation or independently determine treatment. Rather, they are designed to complement conventional clinical, radiologic, and PET/CT assessment by providing an objective estimate of the probability of malignancy before tissue diagnosis. Such information may assist clinicians when considering continued imaging surveillance, additional diagnostic evaluation, or proceeding to surgical resection in patients with indeterminate pulmonary lesions.

The difference in sensitivity and specificity between the radiology/PET and combined models reflects an important clinical trade-off. Although the addition of inflammatory biomarkers increased sensitivity for malignancy detection (0.936 vs. 0.881), this improvement was accompanied by a reduction in specificity (0.852 vs. 0.902). In the setting of cancer surveillance after previous malignancy, false-negative classifications may delay the diagnosis and treatment of recurrent disease, whereas false-positive classifications may lead to unnecessary invasive procedures and procedure-related morbidity. Consequently, the relative preference for the radiology/PET or combined model depends on the clinical context and the relative consequences of these competing errors. When minimizing missed malignancy is the primary priority, the higher sensitivity of the combined model may be preferable. Conversely, when avoiding unnecessary invasive procedures is of greater importance, the higher specificity of the radiology/PET model may represent a more appropriate balance.

In contemporary oncology practice, management decisions for suspicious pulmonary lesions frequently require balancing the risks of delayed cancer treatment against those of unnecessary invasive procedures. The present findings suggest that imaging-centered assessment captures most of the diagnostically actionable information available before tissue confirmation. Consequently, incorporation of multidomain inflammatory biomarker panels may provide limited additional benefit in routine preoperative decision-making. These observations support prioritization of detailed radiologic and metabolic characterization during multidisciplinary evaluation of patients undergoing cancer surveillance.

Another strength of this study lies in its methodological design. To minimize data leakage and better reflect real-world clinical practice, only variables available before surgical decision-making were included in model development. The models were developed using penalized regression with repeated cross-validation and out-of-fold predictions for internal validation, reducing the risk of overfitting. Model performance was assessed from multiple perspectives, including discrimination, calibration, clinical utility, and predictor stability, providing a more comprehensive evaluation than reliance on discrimination alone.

This study has several limitations. First, its retrospective design may have introduced selection bias and residual confounding. Second, because the study population consisted exclusively of patients who underwent surgical resection, the findings may not be generalizable to patients managed non-operatively.

While LASSO regularization was used to minimize overfitting, the relatively low event-to-predictor ratio may still have affected model stability. As a result, the performance of the biomarker and integrated models should be interpreted with caution until validated in larger, independent cohorts. Third, although rigorous internal validation was performed, external validation in independent cohorts is needed before these findings can be generalized. In addition, heterogeneity in primary malignancies, prior treatments, and imaging protocols may have influenced both imaging characteristics and systemic inflammatory profiles. Malignant lesions also could not be systematically subclassified as pulmonary recurrence or pulmonary metastasis because the detailed longitudinal oncologic information required for this distinction was not consistently available in the retrospective dataset. However, review of the histopathological diagnoses confirmed that no cases of second primary lung cancer were identified in the malignant cohort. Furthermore, prior imaging findings and temporal changes in lesion characteristics during oncologic surveillance were not consistently available in a standardized format because of the retrospective study design and were therefore not incorporated into the predictive models. Consequently, the present models were developed using imaging features from the index preoperative assessment rather than longitudinal imaging characteristics. Future prospective studies should evaluate whether serial imaging findings and temporal lesion evolution provide incremental diagnostic value beyond single time-point assessment. Finally, because of the retrospective design, halo sign and reverse-halo sign were recorded as a single combined imaging variable in the study database and therefore could not be analyzed separately. We acknowledge that these represent distinct radiologic findings with different diagnostic implications, and the reverse-halo (atoll) sign is a recognized imaging feature associated with organizing pneumonia. Consequently, combining these findings into a single variable may have reduced the precision of the imaging analysis and limited assessment of their individual diagnostic contributions. Future prospective studies should evaluate halo and reverse-halo signs separately.

Despite these limitations, this study provides a comprehensive comparison of the relative diagnostic contributions of clinical, radiologic/PET, and inflammatory biomarker variables in patients with suspected recurrent pulmonary malignancy [35,36]. The findings consistently indicate that radiologic and PET/CT-derived features account for most of the diagnostic performance, whereas conventional inflammatory biomarkers provide only limited additional information beyond imaging.

Recent studies have also demonstrated the potential of artificial intelligence-assisted quantitative HRCT analysis in thoracic diseases, highlighting the evolving role of advanced imaging analysis in precision diagnostics [37,38]. Future studies should focus on external validation and explore whether incorporating more tumor-specific biological markers, such as radiomic features, circulating tumor DNA, proteomic signatures, or multimodal artificial intelligence approaches, can further improve diagnostic performance.

## 5. Conclusions

In conclusion, integrated preoperative modeling demonstrated excellent performance in distinguishing organizing pneumonia from malignancy in patients with suspected recurrent pulmonary lesions. However, most clinically meaningful predictive information was derived from radiologic and PET/CT features, whereas conventional inflammatory biomarkers provided only limited incremental value. These findings reinforce the central role of imaging in this diagnostic setting and provide a foundation for future imaging-centered translational approaches aimed at improving precision decision-making in thoracic oncology. These findings should be interpreted in the context of a surgically selected cohort and require external validation before being generalized to the broader population of patients undergoing oncologic surveillance.

## Figures and Tables

**Table 1:** Baseline Preoperative Clinical, Radiologic, and Laboratory Characteristics of Patients With Organizing Pneumonia and Malignancy.

Variable		Overall (*n* = 170, %)	Organizing Pneumonia (*n* = 61, %)	Malignancy (*n* = 109, %)	*p*
Clinical	Age (years)	57.5 (48–66)	55 (46–64)	59 (50–68)	0.041
	Sex; Male vs. female	99 (58.2), 71 (41.8)	32 (52.5), 29 (47.5)	67 (61.5), 42 (38.5)	0.285
	ECOG; ≥1 vs. 0	54 (31.8), 116 (68.2)	15 (24.6), 46 (75.4)	39 (35.8), 70 (64.2)	0.147
	Smoking; ever vs. never	85 (50.0), 85 (50.0)	25 (41.0), 36 (59.0)	60 (55.0), 49 (45.0)	0.083
	Comorbidity; yes vs. no	72 (42.4), 98 (57.6)	24 (39.3), 37 (60.7)	48 (44.0), 61 (56.0)	0.574
	Symptomatic; yes vs. no	94 (55.3), 76 (44.7)	28 (45.9), 33 (54.1)	66 (60.6), 43 (39.4)	0.071
Primary tumor origin	Colorectal	74 (43.5)	26 (42.6)	48 (44.0)	0.143
	Breast	29 (17.0)	9 (14.8)	20 (18.3)	
	Genitourinary	27 (15.9)	11 (18.0)	16 (14.7)	
	Gynecologic	16 (9.5)	6 (9.8)	10 (9.2)	
	Other	24 (14.1)	9 (14.8)	15 (13.8)	
Radiological	Lesion diameter (cm)	3.2 (2.1–4.6)	2.6 (1.8–3.9)	3.8 (2.5–5.1)	<0.001
	CT pattern				<0.001
	Solid	122 (71.8)	31 (50.8)	91 (83.5)	
	Ground-glass	14 (8.2)	7 (11.5)	7 (6.4)	
	Consolidation	23 (13.5)	13 (21.3)	10 (9.2)	
	Mixed	11 (6.5)	10 (16.4)	1 (0.9)	
	Cavitation	38 (22.4)	24 (39.3)	14 (12.8)	<0.001
	Spiculation	92 (54.1)	18 (29.5)	74 (67.9)	<0.001
	Air bronchogram	47 (27.6)	22 (36.1)	25 (22.9)	0.081
	Halo sign	29 (17.1)	18 (29.5)	11 (10.1)	0.002
	Multifocality	41 (24.1)	21 (34.4)	20 (18.3)	0.027
	PET-CT SUVmax primary	6.1 (3.9–9.8)	4.2 (2.8–6.5)	7.8 (5.4–12.3)	<0.001
	PET-CT LN involvement	68 (40.0)	14 (23.0)	54 (49.5)	0.001
	PET-CT LN SUVmax	4.5 (2.8–7.2)	2.9 (1.9–4.3)	5.6 (3.6–8.9)	<0.001
Blood-based biomarkers	Hemoglobin	12.8 (11.6–13.9)	12.9 (11.8–14.1)	12.7 (11.5–13.8)	0.362
	NLR	2.93 (1.9–4.1)	2.4 (1.6–3.3)	3.2 (2.2–4.6)	0.014
	MLR	0.49 (0.3–0.7)	0.42 (0.3–0.6)	0.53 (0.4–0.8)	0.027
	PLR	183 (140–245)	165 (130–210)	195 (150–270)	0.036
	PIV	0.62 (0.3–1.1)	0.52 (0.3–0.9)	0.71 (0.4–1.3)	0.042
	SII	827 (520–1320)	680 (450–980)	940 (610–1500)	0.013
	SIRI	2.19 (1.4–3.2)	1.8 (1.2–2.6)	2.5 (1.7–3.6)	0.021
	CAR	0.42 (0.2–0.8)	0.35 (0.2–0.6)	0.48 (0.3–0.9)	0.030
	LAR	4.96 (3.2–7.8)	4.1 (2.9–6.5)	5.6 (3.8–8.9)	0.024
	GINI	0.36 (0.2–0.6)	0.30 (0.2–0.5)	0.40 (0.3–0.7)	0.039

**Abbreviations:** ECOG, Eastern Cooperative Oncology Group; CT, computed tomography; PET/CT, positron emission tomography/computed tomography; SUVmax, maximum standardized uptake value; LN, lymph node; NLR, neutrophil-to-lymphocyte ratio; MLR, monocyte-to-lymphocyte ratio; PLR, platelet-to-lymphocyte ratio; PIV, pan-immune-inflammation value; SII, systemic immune-inflammation index; SIRI, systemic inflammatory response index; CAR, C-reactive protein-to-albumin ratio; LAR, lactate dehydrogenase-to-albumin ratio; GINI, global immune-nutrition-inflammation index. **Footnote**: Data are presented as median (interquartile range) or number (percentage). Group comparisons were performed using the Mann–Whitney U, χ^2^, or Fisher’s exact tests, as appropriate. Only preoperative variables available before surgical decision-making were included in the analyses.

**Table 2:** Discriminative Performance and Diagnostic Classification Metrics of Preoperative Prediction Models.

Model	AUC (95% CI)	Cutoff	Sensitivity	Specificity
Clinical	0.764 (0.682–0.838)	0.670	0.661	0.770
Biomarker	0.778 (0.708–0.839)	0.629	0.697	0.803
Radiology/PET/CT	0.927 (0.877–0.968)	0.613	0.881	0.902
Combined	0.935 (0.895–0.970)	0.539	0.936	0.852

**Abbreviations:** AUC, area under the receiver operating characteristic curve; CI, confidence interval; PET/CT, positron emission tomography/computed tomography. Diagnostic performance of the predefined models for distinguishing malignancy from organizing pneumonia. AUC values are reported with 95% confidence intervals derived from cross-validated out-of-fold predictions. The optimal thresholds were determined using the Youden index. The sensitivity and specificity were reported at this threshold.

## Data Availability

The datasets generated and analyzed in the present study are available from the corresponding author upon reasonable request, contingent upon approval by the Thoracic Surgery Department of the University of Health Sciences Antalya Training and Research Hospital.

## Abbreviations

The following abbreviations are used in this manuscript:

AUC	Area under the receiver operating characteristic curve
CAR	C-reactive protein-to-albumin ratio
CT	Computed tomography
ECOG	Eastern Cooperative Oncology Group
FDG	Fluorodeoxyglucose
GINI	Global immune-nutrition-inflammation index
IQR	Interquartile range
LAR	Lactate dehydrogenase-to-albumin ratio
LASSO	Least absolute shrinkage and selection operator
MLR	Monocyte-to-lymphocyte ratio
NLR	Neutrophil-to-lymphocyte ratio
PET/CT	Positron emission tomography/computed tomography
PIV	Pan-immune-inflammation value
PLR	Platelet-to-lymphocyte ratio
ROC	Receiver operating characteristic
SII	Systemic immune-inflammation index
SIRI	Systemic inflammation response index
SUVmax	Maximum standardized uptake value

## References

[1] Boccatonda A., Guagnano M.T., D’Ardes D., Cipollone F., Vetrugno L., Schiavone C., Piscaglia F., Serra C. (2024). The Role of Contrast-Enhanced Ultrasound in the Differential Diagnosis of Malignant and Benign Subpleural Lung Lesions. J. Clin. Med..

[2] Xu H., Zhu N., Yue Y., Guo Y., Wen Q., Gao L., Hou Y., Shang J. (2023). Spectral CT-Based Radiomics Signature for Distinguishing Malignant Pulmonary Nodules from Benign. BMC Cancer.

[3] Chen H., Stoltzfus K.C., Lehrer E.J., Horn S.R., Siva S., Trifiletti D.M., Meng M.-B., Verma V., Louie A.V., Zaorsky N.G. (2021). The Epidemiology of Lung Metastases. Front. Med..

[4] Nakadate A., Nakadate M., Sato Y., Nakagawa T., Yoshida K., Suzuki Y., Yoshida Y. (2017). Predictors of Primary Lung Cancer in a Solitary Pulmonary Lesion after a Previous Malignancy. Gen. Thorac. Cardiovasc. Surg..

[5] Nadig T.R., Thomas N., Nietert P.J., Lozier J., Tanner N.T., Wang Memoli J.S., Pastis N.J., Silvestri G.A. (2023). Guided Bronchoscopy for the Evaluation of Pulmonary Lesions. Chest.

[6] Tang L., Zhang Y., Wang Y. (2022). Intraoperative Identification of Pulmonary Nodules during Minimally Invasive Thoracic Surgery: A Narrative Review. Quant. Imaging Med. Surg..

[7] Arenas-Jiménez J.J., García-Garrigós E., Ureña Vacas A., Sirera Matilla M., Feliu Rey E. (2022). Organizing Pneumonia. Radiología (Engl. Ed.).

[8] Cherian S.V., Patel D., Machnicki S., Naidich D., Stover D., Travis W.D., Brown K.K., Naidich J.J., Mahajan A., Esposito M. (2022). Algorithmic Approach to the Diagnosis of Organizing Pneumonia. Chest.

[9] Vedachalam S., Freihat M., Alomari A.K., Sears C.R., Al Nasrallah N. (2026). The Concurrence of Lung Malignancies and Organizing Pneumonia. Front. Oncol..

[10] Radzikowska E., Fijolek J. (2023). Update on Cryptogenic Organizing Pneumonia. Front. Med..

[11] Sakthivel M.K., Hazelton T.R., Askin F.B., Bantumilli S., Sridhar S. (2026). Organizing Pneumonia Phenotype. Semin. Roentgenol..

[12] Kim C., Liberman Y., Borisovsky G., Litmanovich D., VanderLaan P., Brook A., Brook O.R. (2025). Incidence of Malignancy in Lung Lesions Initially Classified as Organizing Pneumonia on CT-Guided Biopsies. Abdom. Radiol..

[13] Tang W., Wu J., Yang S., Wang Q., Chen Y. (2022). Organizing Pneumonia With Intense 68Ga-FAPI Uptake Mimicking Lung Cancer on 68Ga-FAPI PET/CT. Clin. Nucl. Med..

[14] Cook A.E., Garrana S.H., Martínez-Jiménez S., Rosado-de-Christenson M.L. (2022). Imaging Patterns of Pneumonia. Semin. Roentgenol..

[15] Pritzker K.P.H. (2023). Blood-Based Biomarkers of Chronic Inflammation. Expert Rev. Mol. Diagn..

[16] Tian B.-W., Yang Y.-F., Yang C.-C., Yan L.-J., Ding Z.-N., Liu H., Xue J.-S., Dong Z.-R., Chen Z.-Q., Hong J.-G. (2022). Systemic Immune–Inflammation Index Predicts Prognosis of Cancer Immunotherapy: Systemic Review and Meta-Analysis. Immunotherapy.

[17] Leng J., Wu F., Zhang L. (2022). Prognostic Significance of Pretreatment Neutrophil-to-Lymphocyte Ratio, Platelet−to−Lymphocyte Ratio, or Monocyte-to-Lymphocyte Ratio in Endometrial Neoplasms: A Systematic Review and Meta−analysis. Front. Oncol..

[18] Hajibandeh S., Hajibandeh S., Romman S., Parente A., Laing R.W., Satyadas T., Subar D., Aroori S., Bhatt A., Durkin D. (2023). Preoperative C-Reactive Protein-to-Albumin Ratio and Its Ability to Predict Outcomes of Pancreatic Cancer Resection: A Systematic Review. Biomedicines.

[19] Cetintepe T., Cetintepe L., Guc Z., Ertekin I., Karaalp O., Alacacioglu A. (2025). LDH-to-Albumin Ratio (LAR) in Solid Tumor Patients with Cytopenia: A Simple Biomarker to Predict Bone Marrow Metastasis. J. Clin. Med..

[20] Aydin A.A., Yuceer R.O. (2024). Unraveling the Predictive Value of the Novel Global Immune-Nutrition-Inflammation Index (GINI) on Survival Outcomes in Patients with Grade 4 Adult-Type Diffuse Gliomas. Curr. Oncol..

[21] Prajumwongs P., Titapun A., Thanasukarn V., Jareanrat A., Khuntikeo N., Namwat N., Klanrit P., Wangwiwatsin A., Chindaprasirt J., Koonmee S. (2025). Identification of Serum Metabolite Biomarkers and Metabolic Reprogramming Mechanisms to Predict Recurrence in Cholangiocarcinoma. Sci. Rep..

[22] Perwaiz M., Latif K., White A., Khalid A. (2026). Blood-Based Biomarkers for Pulmonary Nodule Risk Stratification. J. Bronchol. Interv. Pulmonol..

[23] Adelsmayr G., Janisch M., Müller H., Holzinger A., Talakic E., Janek E., Streit S., Fuchsjäger M., Schöllnast H. (2023). Three Dimensional Computed Tomography Texture Analysis of Pulmonary Lesions: Does Radiomics Allow Differentiation between Carcinoma, Neuroendocrine Tumor and Organizing Pneumonia?. Eur. J. Radiol..

[24] Gong J., Zhang Z., Luo T., Huang X., Zhu C., Lv J., Li Q. (2022). Combined Model of Radiomics, Clinical, and Imaging Features for Differentiating Focal Pneumonia-like Lung Cancer from Pulmonary Inflammatory Lesions: An Exploratory Study. BMC Med. Imaging.

[25] Erinanc H., Uçar Y., Karabulut K.U., Yonar A., Unler A.G.K., Kulaksizoglu S. (2025). Role of Systemic Inflammatory Markers in the Differential Diagnosis of Organizing Pneumonia and Lung Cancer. APMIS.

[26] Aminu M., Daver N., Godoy M.C.B., Shroff G., Wu C., Torre-Sada L.F., Goizueta A., Shannon V.R., Faiz S.A., Altan M. (2023). Heterogenous Lung Inflammation CT Patterns Distinguish Pneumonia and Immune Checkpoint Inhibitor Pneumonitis and Complement Blood Biomarkers in Acute Myeloid Leukemia: Proof of Concept. Front. Immunol..

[27] Hai-Jing Y., Shan R., Jie-Qiong X. (2023). Prognostic Significance of the Pretreatment Pan-Immune-Inflammation Value in Cancer Patients: An Updated Meta-Analysis of 30 Studies. Front. Nutr..

[28] Zhang S., Cheng T. (2024). Prognostic and Clinicopathological Value of Systemic Inflammation Response Index (SIRI) in Patients with Breast Cancer: A Meta-Analysis. Ann. Med..

[29] Chu Z., Sheng B., Liu M., Lv F., Li Q., Ouyang Y. (2016). Differential Diagnosis of Solitary Pulmonary Inflammatory Lesions and Peripheral Lung Cancers with Contrast-Enhanced Computed Tomography. Clinics.

[30] Archer J.M., Mendoza D.P., Hung Y.P., Lanuti M., Digumarthy S.R. (2023). Surgical Resection of Benign Nodules in Lung Cancer Screening: Incidence and Features. JTO Clin. Res. Rep..

[31] Qiao K., Qin X., Fu S., Ren J., Jia J., Hu X., Tao Y., Yuan S., Wei Y. (2023). Value of [18F]AlF-NOTA-FAPI-04 PET/CT for Differential Diagnosis of Malignant and Various Inflammatory Lung Lesions: Comparison with [18F]FDG PET/CT. Eur. Radiol..

[32] Puuvuori E., Liggieri F., Velikyan I., Chiodaroli E., Sigfridsson J., Romelin H., Ingvast S., Korsgren O., Hulsart-Billström G., Perchiazzi G. (2022). PET-CT Imaging of Pulmonary Inflammation Using [68Ga]Ga-DOTA-TATE. EJNMMI Res..

[33] Korkmaz F.T., Traber K.E. (2023). Innate Immune Responses in Pneumonia. Pneumonia.

[34] Van Calster B., Steyerberg E.W., Wynants L., van Smeden M. (2023). There Is No Such Thing as a Validated Prediction Model. BMC Med..

[35] Pessoa L.S., Heringer M., Ferrer V.P. (2020). CtDNA as a Cancer Biomarker: A Broad Overview. Crit. Rev. Oncol. Hematol..

[36] Li K., Liu K., Zhong Y., Liang M., Qin P., Li H., Zhang R., Li S., Liu X. (2021). Assessing the Predictive Accuracy of Lung Cancer, Metastases, and Benign Lesions Using an Artificial Intelligence-Driven Computer Aided Diagnosis System. Quant. Imaging Med. Surg..

[37] Russo A., Patanè V., Oliva A., Viglione V., Franzese L., Forte G., Liakouli V., Perrotta F., Reginelli A. (2025). AI-Based HRCT Quantification in Connective Tissue Disease-Associated Interstitial Lung Disease. Diagnostics.

[38] Russo A., Patanè V., Ruotolo F., Brunese M.C., Del Canto M., Alessio L., Monari C., Coppola N., Reginelli A. (2026). Artificial Intelligence-Assisted Quantification of Longitudinal HRCT Changes During Treatment of Pulmonary Tuberculosis: An Exploratory Proof-of-Concept Study. Diagnostics.

